# Metabolic Dysfunction-associated Steatotic Liver Disease and Type 2 Diabetes: A Deadly Synergy

**DOI:** 10.17925/EE.2024.20.2.2

**Published:** 2024-04-23

**Authors:** Damien Leith, Yeun Yi Lin, Paul Brennan

**Affiliations:** Clinical Research Centre, Ninewells Hospital, Dundee, UK

**Keywords:** Alcohol-related liver disease, cardiometabolic risk factors, metabolic dysfunction-associated steatotic liver disease, metabolic and alcoholic-related steatotic liver disease, metabolic dysfunction-associated steatohepatitis, non-alcoholic fatty liver disease, steatotic liver disease, type 2 diabetes, liver cirrhosis, non-alcoholic steatohepatitis

## Abstract

Type 2 diabetes (T2D) and metabolic dysfunction-associated steatotic liver disease (MASLD) are both facets of the metabolic syndrome, associated with obesity and insulin resistance. MASLD, a term that replaces non-alcoholic fatty liver disease (NAFLD), occurs in up to 70% of people with T2D. Not only do T2D and MASLD commonly co-occur, but there is a synergistic, bidirectional relationship between these conditions, meaning that each affects the natural disease course of the other. As such, it is important for those caring for people with T2D to recognize the importance of this co-diagnosis. In this summary, we detail the synergistic relationship between T2D and MASLD, explain the current challenges in recognizing this common co-diagnosis and suggest practical approaches for those caring for people with T2D to improve the diagnosis and treatment of MASLD.

Type 2 diabetes (T2D) continues to pose an ever-greater global health challenge, with 1.31 billion individuals predicted to be living with diabetes globally by 2050; the majority of whom will have T2D.^1^ Closely linked to T2D is metabolic dysfunction-associated steatotic liver disease (MASLD); defined as liver steatosis, identified on biopsy or imaging, in the presence of at least one other cardiometabolic risk factor (CMRF).^2^ This definition replaced non-alcoholic fatty liver disease (NAFLD), which was felt to be stigmatizing.^2,3^ The new definition of MASLD differs importantly from that of NAFLD in being a positive diagnosis, which emphasizes metabolic dysfunction as its cause, distinct from NAFLD that only emphasized what the condition was not (i.e. non-alcoholic). This positive MASLD definition also allows for the dual aetiology of MASLD with other causes of liver steatosis or liver disease (e.g. MASLD plus drug-i nduced steatosis or MASLD plus autoimmune hepatitis). MASLD sits within the new broad grouping of steatotic liver disease and along with metabolic and alcohol-related steatotic liver disease (MetALD) and alcohol-related liver disease (ALD), forms part of a continuum of conditions differentiated by alcohol intake (see Figure 1). Importantly, this new terminology accounts for the frequent co-existence of metabolic dysfunction with moderate or greater alcohol intake, previously excluded by NAFLD criteria.

MASLD encompasses a spectrum of disease from simple steatosis, through metabolic dysfunction-associated steatohepatitis (MASH), without or with fibrosis, to compensated and, finally, decompensated cirrhosis and hepatocellular carcinoma (HCC). It is important to note that, whilst the NAFLD and MASLD definitions are similar, updated studies will be required into aspects of MASLD, including its prevalence, progression and treatment, to fully understand how this may differ from NAFLD. However, early evidence suggests significant overlap, and in this article, studies undertaken using the NAFLD criteria will be considered to apply to MASLD.^4^ In addition, for clarity the term MASLD will be used throughout this article, even when describing studies that took place using the previous NAFLD criteria.

MASLD is seeing a rapid increase in prevalence – modelling based on current trajectories suggests the current global prevalence of MASLD in adults is approaching 40% and is set to increase to 55% of adults globally by 2040.^5–7^ This coincides with a two to threefold increase in the most severe forms of the disease and its complications, including decompensated liver cirrhosis, HCC and death.^8^ MASLD has overtaken ALD as the leading cause of incident liver cirrhosis and is likely to become the leading cause of liver-related mortality and transplantation in the coming decades.^9–11^

## Why is it important to recognize this association?

Both T2D and MASLD are aspects of metabolic syndrome, share overlapping pathophysiological mechanisms and, as such, they often co-exist.^12,13^ Both MASLD and T2D occur due to a complex inter-relationship between genetic propensity, over nutrition, poor diet and reduced physical exercise resulting in obesity, with associated chronic low-grade inflammation and insulin resistance.^12,13^ Whilst obesity is an important cofactor in MASLD and T2D, a proportion of people have so called 'lean disease', that is, MASLD or T2D in individuals with a BMI <25 kg/m^2^ in non-Asian or BMI<23 kg/m^2^ in Asian individuals.^14,15^ In these people, disproportion adipose distribution (tending towards central and visceral adiposity) and adipose dysfunction, sarcopaenia and early beta cell failure are likely to be significant drivers of MASLD and T2D development.^12–14^

**Figure 1:**
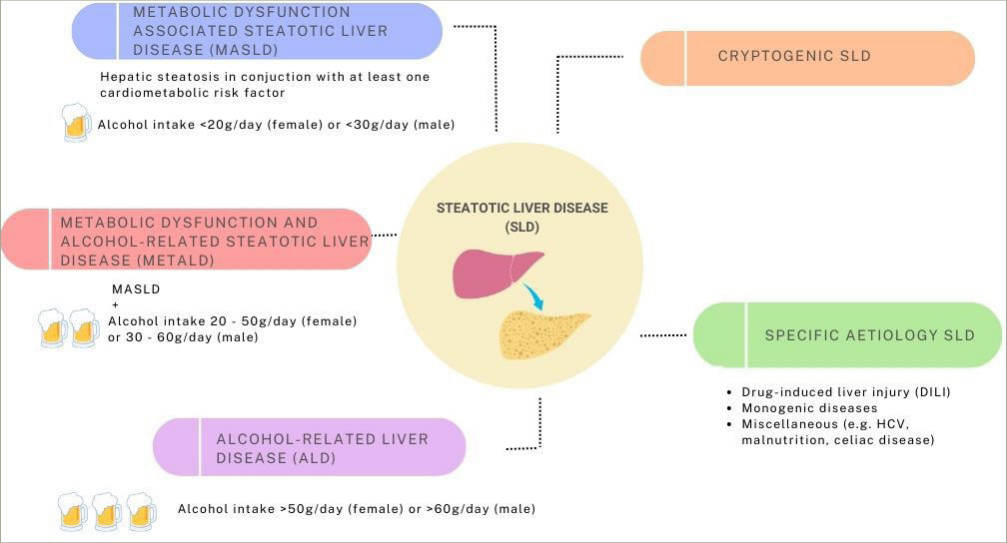
Aetiologies of steatotic liver disease

*ALD = alcohol-related liver disease; DILI = drug-induced liver injury; HCV = hepatitis C virus; MASLD = metabolic dysfunction-associated steatotic liver disease; MetALD = metabolic dysfunction and alcohol related steatotic liver disease; SLD = steatotic liver disease.*

As a result of the linked pathophysiologies, MASLD is extremely common in patients with T2D, with prevalence demonstrated to be as high as 65–70%, and studies using liver biopsy-based diagnosis demonstrating prevalence of over 90%.^16–18^ In addition, there is a bi-directional, synergistic interaction between the conditions, which leads to worsening diabetes control, increased risk of MASLD progression and increased non-l iver morbidity and mortality beyond that already conferred by T2D alone.

It is therefore essential for those caring for patients with T2D to be aware of the high probability of comorbidity with MASLD, understand its implications for patients with T2D and recognize how this co-diagnosis may change T2D treatment approaches.

### The impact of metabolic dysfunction-associated steatotic liver disease on type 2 diabetes

MASLD has been demonstrated to be a pre-diabetic condition; patients with MASLD have a 2–5 times higher risk of incident T2D than that of the general population, with the risk increasing with severity of liver fibrosis.^19–21^ MASLD increases peripheral and hepatic insulin resistance, and therefore hyperinsulinaemia. Furthermore, individuals with T2D and MASLD, especially those with fibrosis have worse glycated haemoglobin (HbA1c) control over time compared with those without MASLD.^22,23^

### The impact of type 2 diabetes on metabolic dysfunction-associated steatotic liver disease

Reciprocally, T2D is associated with a worse MASLD phenotype. Patients with MASLD and T2D have a higher likelihood of progressive fibrosis and faster rate of fibrosis progression than those without.^24^ As a result, more individuals progress to liver cirrhosis, cirrhosis decompensation and have a higher risk of liver-related mortality.^25,26^ In prospective cohorts of patients with T2D not known to have MASLD, screening has demonstrated rates of advanced liver fibrosis of 14% and cirrhosis of 3–6%.^16,17^ Furthermore, T2D increases the risk of HCC twofold and risk of death from HCC 1.5-fold in patients with MASLD compared with MASLD without T2D.^27^

### Comorbidities associated with the combination of type 2 diabetes and metabolic dysfunction-associated steatotic liver disease

The combination of T2D and MASLD also significantly increases the risk of non-l iver morbidity and mortality, and cardiovascular disease and non-liver malignancy remains the leading causes of death in patients with MASLD, ahead of liver-related mortality, in those with and without T2D.^28^ Patients with T2D and MASLD have an increased risk of cardiovascular disease (CVD), arrhythmia and chronic kidney disease progression compared with those without MASLD.^29,30^ Patients with T2D and MASLD or liver fibrosis (based on non-i nvasive testing) also have increased cardiovascular and all-cause mortality compared with those with T2D without MASLD.^26,31^

## Challenges in diagnosing metabolic dysfunction-associated steatotic liver disease

Diagnosis of MASLD cirrhosis is often late. The majority of patients’ index presentations are in secondary care with symptoms of advanced liver disease, clearly demonstrating the scope for earlier diagnosis of MASLD, and identification of those who are at risk of developing advanced liver disease.^32^ This delay in diagnosis is driven by the diagnostic challenges of MASLD, including the poor sensitivity of routine biochemical tests and the lack of proactive screening approaches for case detection, in addition to a lack of patients' and clinicians’ awareness of the importance of MASLD.

Patients with MASLD are usually asymptomatic before the development of decompensated cirrhosis.^33^ Therefore, diagnosis of MASLD relies on a high index of clinical suspicion and biochemical or radiological testing. Routine liver function tests (LFTs) are performed very commonly in the general population and often performed regularly among the population with diabetes.^34^ However, LFT cut-off values are ineffective to detect MASLD with advanced fibrosis or cirrhosis, and 30% of cases may still be missed despite lowering cut-off values to below current reference ranges.^35,36^

Recent guidelines now recommend assessing for MASLD fibrosis in all patients with T2D.^33,37,38^ This should be achieved using non-i nvasive (NI) tests with high negative predictive value aimed at 'ruling out' fibrosis. The Fibrosis-4 (FIB-4) score, which is simple to calculate from routine tests requested during clinical practice, is probably the most widely known and validated.^39,40^ This has a higher sensitivity than alanine transaminase (ALT) alone to identify patients with MASLD and liver fibrosis.^16,35^ Patients with FIB-4 scores ≥1.30 (aged 36–64 years) or ≥2.0 (aged >65 years) should be assessed further, with the caveat that FIB-4 has not been validated for those <35 years. However, there is evidence that FIB-4 has a lower sensitivity to detect advanced fibrosis in patients with T2D than in the general population.^41^ Hence, negative FIB-4 should not preclude a hepatology review in cases where a clinician has a high index of suspicion or alternative tests suggest possible fibrosis. Annual screening with FIB-4 may increase the chance of picking up those patients who have had falsely reassuring FIB-4 results previously.^42^ The use of sequential non-invasive modalities improves overall performance in identifying those with likely advanced fibrosis, although evidence of utility in exclusively T2D populations is lacking.^43^

### Patients’ and clinicians’ lack of awareness

Despite the increasing global prevalence of MASLD, studies show as few as 3.1% of people at risk of MASLD are aware of the diagnosis, with another study showing only 2.4% of patients with radiologically confirmed MASLD, a third of whom had diabetes, were aware of their diagnosis.^44,45^

Historically, the lack of national guidelines for LFT interpretation, particularly in the community healthcare settings, along with the limitations of current ‘normal’ reference range for assays, may have led to the potential overlooking of results that fall within the ‘non-significantly abnormal range’.^46^ Additionally, the situation can be exacerbated by physicians’ hesitancy to address the condition due to a perceived lack of confidence discussing the condition, time constraints in clinics and preference to avoid overwhelming their patients.^47^ These factors are likely to contribute to the patients’ lack of insight into their conditions.

## Implication: what you can do (time to act!)

### Improving screening

The improved detection of MASLD in patients with T2D, especially in those with fibrosis, is essential. Detecting early fibrosis enables targeted risk factor modification to prevent disease progression and development of complications.^48^

MASLD screening should be incorporated into diabetes annual reviews, with all patients with T2D having a FIB-4 calculated every 1–2 years, regardless of LFT result.^38^ For those with indeterminate or high-risk scores, or those with a normal FIB-4 but persistently elevated LFTs, further non-i nvasive testing should be undertaken, such as enhanced liver fibrosis (ELF) score or Fibroscan® (Echosens, Paris, France) where available.^49^ Patients with second-l ine test results suggestive of advanced fibrosis, or in settings where there is no easy access to these tests, should be referred to hepatology services for further specialist input.

### Targeted lifestyle modification

Lifestyle modification remains a cornerstone of care in both T2D and MASLD. Weight loss is associated with a reduction in liver enzymes, radiological steatosis, liver stiffness and histological NAFLD improvement, with the degree of improvement related to the amount of weight lost.^50^ Patients should be encouraged that benefit has been seen with weight loss ≥5%, reduction in inflammation seen in all patients with weight loss of 7–10% and further improvements, including increased regression of histological fibrosis with weight loss >10%.^51^ Weight loss is difficult to achieve and harder to maintain, and patients should be aided with individualized weight-l oss targets (aiming for gradual, sustained loss of 7–10%) and referral to assisted weight-l oss programmes where available.

Multiple studies have assessed the aetiological role and potential therapeutic benefits of different dietary macro and micronutrient compositions, specific food types, patterns of eating (e.g. intermittent fasting) and regional diets (e.g. the Mediterranean diet), as treatments in patients with MASLD.^52,53^ Calorie intake has been shown to be the most important dietary factor in the development of MASLD in a systematic review and calorie restriction is a beneficial treatment in MASLD, and also to be highly effective in the management of T2D.^52,54^ Both the American Association for the Study of Liver Disease and the American Diabetes Association recommend the Mediterranean diet due to its benefits in reducing cardiovascular risk and possibly liver steatosis.^33,55^

### Treatment selection

Whilst there are currently no licensed treatments for MASLD, several diabetes treatments have good evidence of efficacy in reducing liver fat and inflammation in patients with MASLD, and should be considered for patients with co-existing T2D and MASLD.^56^

#### Glucagon-like peptide-1 receptor agonists

Semaglutide, dulaglutide, liraglutide (1.8 mg/day), exenatide and lixisenatide have been shown to be effective in reducing steatohepatitis, compared with controls and glucagon-l ike peptide-1 (GLP-1) receptor agonists has the additional benefit of aiding weight loss.^57–60^

#### Thiazolidinediones

Pioglitazone, a thiazolidinedione, has also been demonstrated in placebo-controlled biopsy studies in patients with diabetes to be an effective long-term treatment to reduce steatosis and inflammation in MASLD.^61^ Care should be taken in patients at risk of heart failure and pioglitazone is also associated with weight gain.^62^

#### Sodium-glucose cotransporter-2 inhibitors

Sodium-glucose cotransporter-2 inhibitors (SGLT-2i) have not been studied in paired biopsy studies in MASLD, however multiple placebo or active-controlled studies, utilizing reduction in serum liver enzyme levels or liver fat content on imaging as end points, have been undertaken. A meta-analysis of these studies (in which 90% of the patients had T2D) demonstrated SGLT-2i were effective at reducing liver enzymes (ALT and gamma-glutamyltransferase [GGT]) and liver fat content measured with magnetic resonance imaging (MRI), suggesting they may have utility in treating steatohepatitis associated with MASLD.^63^

Conversely metformin, sulphonylureas, dipeptidyl peptidase-4 (DPP4) inhibitors and insulin have not been demonstrated to have additional benefit in the treatment of MASLD beyond improvement in diabetes control and should not be preferred treatments for patients with T2D and MASLD if the above treatments could be used in the first instance.^12,64^

### Emerging therapies

Evidence is emerging that newer T2D treatments may also have activity in MASLD. Post hoc analysis of the phase II trial of tirzepatide, a dual GLP-1 and glucose-dependent insulinotropic polypeptide (GIP) receptor agonist, showed a significant improvement in adinopectin level and other MASLD-related biomarkers in participants taking higher doses of tirzepatide, compared with placebo.^65^ In a separate sub-study of a phase III trial, tirzepatide was shown to reduce liver steatosis on MRI compared with participants who received insulin degludec.^66^ Further MASLD-specific studies on the efficacy of tirzapetide are necessary to support its clinical use in the management of MASLD.

Given the frequent overlap of T2D and MASLD, it will be important to ascertain early if emerging T2D therapies have efficacy in MASLD to allow clinicians to offer patients with this dual diagnosis the most effective therapy.

### Metabolic-bariatric surgery

Metabolic surgery (such as sleeve gastrectomy or Roux-en-Y gastric bypass) in selected patients has also been demonstrated to be effective in treating MASLD by improving steatosis and inflammation in patients with MASLD (as well as aiding weight loss and improving HbA1c and insulin resistance) and may improve liver fibrosis.^67^ Whilst MASLD alone is not an indication for metabolic surgery, it could constitute an additional benefit when considering this treatment option for high-risk patients with T2D and obesity, particularly in the presence of fibrosis. The current evidence to support metabolic surgery in patients with cirrhosis is less clear and it is not recommended currently due to risk of decompensation and excess mortality.^68^

### Multidisciplinary involvement

Primary care physicians and diabetologists should have the confidence to refer their patients to specialist liver clinics, where hepatologists can be involved in patients’ care early when advanced fibrosis or cirrhosis is suspected. This enables timely detections of potential complications, such as varices and HCC, enabling prompt treatment. In a T2D population-based study examining the prevalence of MASLD, the integration of a clear specialist hepatology services referral pathway resulted in liver clinic appointments for approximately 5% of study participants.^69^ While this percentage may appear modest, it is notably significant considering the increasing prevalence of T2D. This approach successfully identified previously undiagnosed cases of cirrhosis and HCC.^69^

The advent of multidisciplinary MASLD clinics has been proposed given the multimorbid nature of the condition, whereby patients can engage with relevant medical specialists and allied health professionals.^70^ However, this is resource intense, and challenging to deliver. It is, however, important to recognize that there needs to be a widening of the stakeholders involved in tackling MASLD to instigate meaningful change.^71^

## Conclusion

T2D and MASLD represent overlapping global health emergencies linked to increasing rates of global obesity and metabolic syndrome.^13^ There is a synergistic interaction between the two conditions, resulting in poorer outcomes. Despite a prevalence of up to 70%, MASLD is underdiagnosed in patients with T2D, meaning presentation is often late, at a stage when there is limited scope for treatment.^16–18,32^ Those caring for patients with T2D, such as diabetologists, are in an important position to help identify MASLD early. All patients with T2D should be screened with FIB-4 at least every 2 years, with this screening incorporated into routine diabetic check-ups.^38^ Those identified at indeterminate or high risk for MASLD with fibrosis should undergo additional evaluation.^49^ For patients with T2D identified to have MASLD, this should be explained to the patient, as most will not be aware of MASLD (*Figure S1*).^45^

The clear benefit of weight loss on both patients with T2D and MASLD (aiming for a 7–10% reduction) should be stressed and assistance offered in the form of weight management service referral and, in selected patients, consideration of metabolic surgery.^51,67^ When choosing treatments for T2D in patients with MASLD, opting for treatments with proven additional benefit in MASLD (pioglitazone, GLP1 receptor agonists and SGLT2i) is advised. Finally, close collaboration with hepatology colleagues and early referral for patients with advanced fibrosis or cirrhosis is essential. The availability of novel pharmacotherapy for MASLD is dependent on more human-centric approaches in drug development.^72^ Until then, optimization of metabolic dysfunction using currently licensed drugs remains pivotal in halting disease progression and turning the tide on this deadly synergy, and public health epidemic.
